# Peri-urban area delineation and urban sprawl quantification in Thiruvananthapuram Urban Agglomeration, India, from 2001 to 2021 using geoinformatics

**DOI:** 10.1007/s12518-022-00460-0

**Published:** 2022-08-22

**Authors:** Vishal Chettry

**Affiliations:** Manipal School of Architecture and Planning, Manipal Academy of Higher Education, Manipal, India

**Keywords:** Peri-urban areas, Urban sprawl, Midsized city, Thiruvananthapuram, India

## Abstract

In Southeast Asia, rising population, economic growth, and lack of land supply in the city core have led to the widespread irreversible land cover transformation in peri-urban areas. Such extensive and haphazard urban growth in peri-urban areas raises concern about analyzing and promoting planned urban growth. Therefore, this paper attempts to assess land cover changes from 2001 to 2021 and delineate peri-urban areas of a midsized Indian city, i.e., Thiruvananthapuram Urban Agglomeration (UA) using geoinformatics. The maximum likelihood supervised classification tool in ArcGIS 10.3 was used to prepare land cover maps for 2001, 2007, 2014, and 2021. Further, the presence of urban sprawl in the peri-urban areas was detected through Shannon’s entropy index. The urban sprawl typologies in the peri-urban areas were quantified using the adjacent neighborhood relationships concept. The results revealed rapid growth in built-up land cover and decline in non-built-up land cover within Thiruvananthapuram UA during the study period. Peri-urban areas were delineated based on nine indicators, such as total population, population growth, population density, literacy rate, percentage of the male workforce, percentage of the female workforce, agricultural land cover, distance from urban core, and percentage of cultivators to agricultural workers. A rise in Shannon’s entropy index from 1.59 in 2001 to 2.05 in 2021 exhibited the occurrence of rapid urban sprawl in the peri-urban areas. Dominance of discontinuous low-density development, i.e., scatter development typology of urban sprawl, was observed in peri-urban areas of Thiruvananthapuram UA. Such studies using geoinformatics would assist local governments in scientifically formulating sustainable urban policies and plans.

## Introduction

Globally, industrialization has resulted in the land transformation from rural to urban, also termed urbanization (Tian et al. 2017; Basu et al. 2020). Due to urbanization, a reduction in poverty, increased literacy rate, and higher employment opportunities have occurred in most Asian countries (Roberts and Kanaley 2006). However, urbanization has marked irreversible land cover changes over the few decades, primarily due to intense anthropogenic activities and the absence or weak implementation of regulations and infrastructural developments (Mishra et al. 2018). Such urban growth phenomenon has led to horizontal expansion of urban areas to nearby census towns (CTs) and outgrowths (OGs), as identified during the Indian census survey (Dutta and Das 2019a). As a consequence, the gap between urban and rural features has narrowed, and a rise in mixed land use, often termed as desakota regions or peri-urban areas, is observed (Dadashpoor and Ahani 2019).

Peri-urban areas are the settlements located surrounding the core cities, wherein both urban and rural activities coexist, and the land cover is prone to quick transformations by intense anthropogenic activities (Douglas 2006). However, much ambiguity in this domain remains related to definitions, characteristics, typologies, and policy-making and implementation (Amirinejad et al. 2018). Due to increased proximity, urban and peri-urban areas are strongly interdependent for facilities and services. Moreover, the population growth and unplanned development of the cities in the form of urban sprawl engulf the land in peri-urban areas within the municipal boundaries (Vishwanath et al. 2013; Kar et al. 2018). Urban sprawl is an unsustainable form of development due to poor utilization of land resources and is a major challenge in peri-urban areas (Gonçalves et al. 2017). Such extensive and haphazard development in peri-urban areas raises the concern to analyze and plan for urban sustenance. In this context, the United Nations 2030 Agenda for Sustainable Development (SDG 11) suggests that each city adopts sustainable urban planning practices to promote sustainable cities and communities (United Nations 2015; Samat et al. 2021).

There are numerous techniques adopted to delineate peri-urban areas. For this purpose, spatial metrics and socioeconomic variables such as population size, population density, infrastructural availability, administrative boundary, and major economic base characterize peri-urban areas (Gonçalves et al. 2017). Another approach includes the use of population density and distance to existing urban centers (Reginster and Rounsevell 2006; Piorr et al. 2011; Nishara et al. 2021). Census datasets are also used to identify peri-urban areas (Mondal 2021; Coluzzi et al. 2022). Others utilize land cover change patterns to delineate peri-urban areas (Brinkmann et al. 2012; Mortoja and Yigitcanlar 2021). Remote sensing (RS) imageries, in combination with GIS technology, have been immensely used to analyze peri-urban areas (Gazi et al. 2020; Abd El-Hamid et al. 2021; Boakye et al. 2020). Due to the difference in socioeconomic and ecological features of peri-urban areas, the transformation of rural to urban has a profound impact on the region. The urban growth pattern in the peri-urban areas has caused a shift in the function of urbanity to the fringes, thus promoting urban sprawl (Hidajat et al. 2013; Schneider et al. 2014). Hence, this study attempts to delineate peri-urban areas and analyze urban sprawl.

Earle Draper first mentioned the term urban sprawl in his speech at a national conference of planners in 1937 to describe its undesirable effects on society and the economy (Wassmer 2002; Ismael 2020). Post-1961, the concept of urban sprawl gained momentum after Jane Jacobs published her famous essay titled “The death and life of great American cities.” Urban sprawl is considered a random and haphazard low-density urban growth pattern that eventually contributes to inefficient usage of resources (Ewing 1997; Galster et al. 2001; Bhatta 2010; Kashem et al. 2014; Kamruzzaman et al. 2018). The immediate consequence of urban sprawl is a transformation in the land cover of an area, mostly due to a rise in the built-up and impervious surfaces (Morya and Ram 2020; Chettry and Surawar 2021a, b, c). RS has been effectively used in the urban sprawl research to detect urban sprawl and map the temporal patterns of urban sprawl, e.g., Guangzhou, China (Yu and Ng 2007); Europe (Arribas-bel et al. 2011); Italy (Nol et al. 2014); Teresina, Brazil (Espindola et al. 2017); Western Cape Province, South Africa (Horn and Eeden 2018); and Vijayawada, India (Vani and Prasad 2020). Landscape metrics are based on information theory and fractal geometry concepts to characterize the urban sprawl (Wang et al., 2018). Entropy indexes such as Shannon’s entropy Index, relative entropy index, and structure entropy index are used to assess the intensity of urban sprawl (Jiang et al. 2016; Lemoine-Rodríguez et al. 2019). Further, urban sprawl can be categorized into infill development, edge-expansion, and outlying growth based on the pattern of new built-up units surrounding the old built-up (Chettry and Surawar 2021a). In India, the large cities are exhibiting a decline in the population growth rate, primarily due to surpass in their carrying capacity. Thus, intense urban growth is forecasted to occur in mid-sized cities, also known as Tier-II cities (population between 0.5 to 5 million) (Mitra and Mehta 2011; Perez et al. 2019) . However, research on urban sprawl assessment in peri-urban areas of Indian mid-sized cities is relatively scarce.

The spatial boundary of Thiruvananthapuram Urban Agglomeration (UA) has tremendously increased from 256.22 km^2^ in 2001 to 542.57 km^2^ in 2011, with a rate of 112% within a decade. The Census of India notifies UA based on the population growth in outgrowths adjoining any municipal area (Census of India 2011). This administrative unit is most appropriate for the study because it encompasses nearby peri-urban areas, where urban sprawl is likely to occur. Therefore, this study attempts to delineate peri-urban areas and assess urban sprawl in Thiruvananthapuram UA from 2001 to 2021, using geoinformatics. The primary objectives are to (1) conduct land cover change detection in Thiruvananthapuram UA, (2) delineate peri-urban areas as per the derived index, (3) detect and analyze urban sprawl in the peri-urban areas, and (4) quantify urban sprawl typologies. Such studies would assist in visualizing and understanding the urban sprawl dynamics in the peri-urban areas and promote sustainable urbanization.

## Study area and data sources

**Fig. 1:**
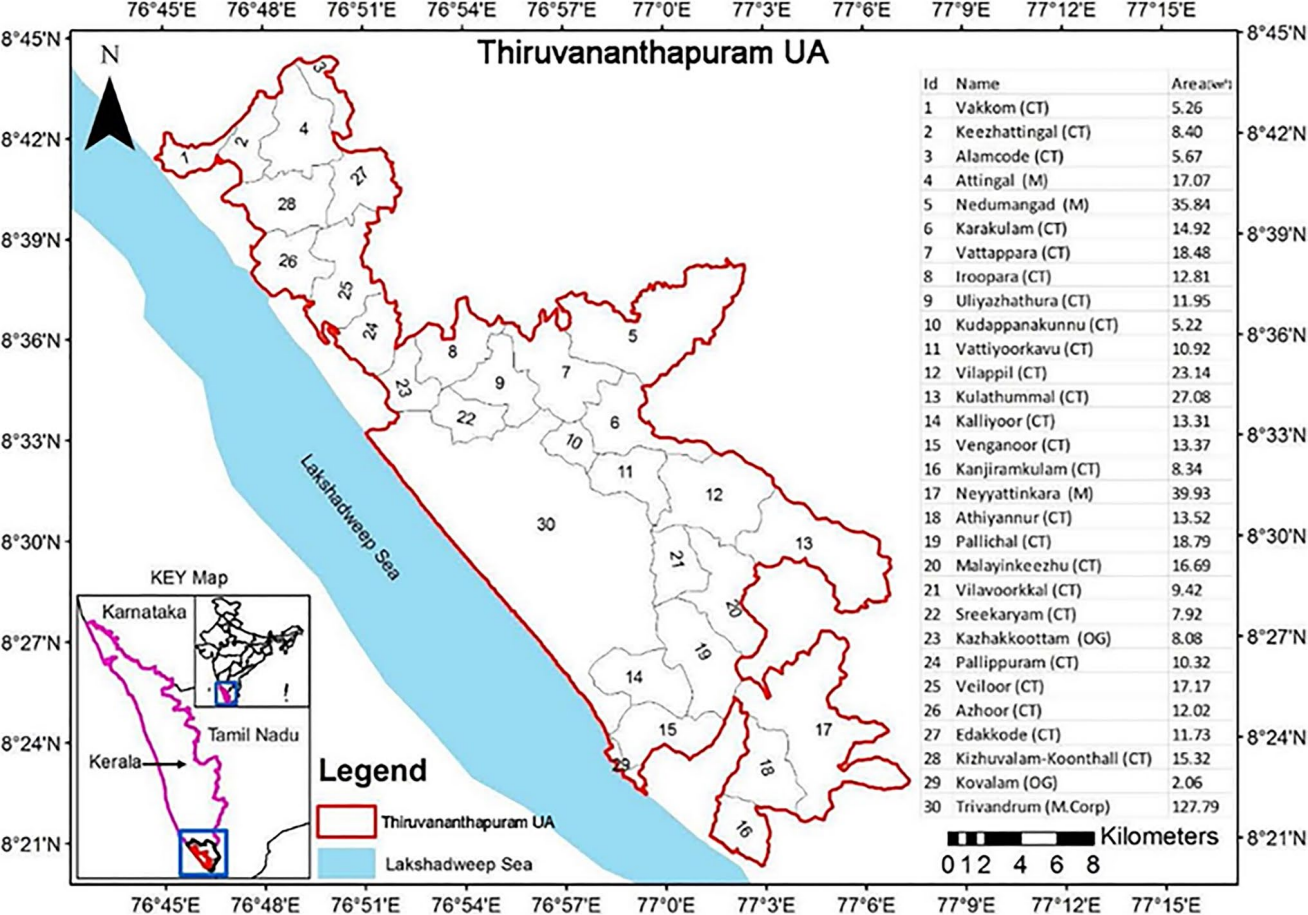
Location map of Thiruvananthapuram Urban Agglomeration

Thiruvananthapuram UA is situated between 76°45′ E–77°8′ E longitudes and 8°45′ N–8°21′ N latitudes, wherein the elevation varies from 0 to 257 m above mean sea level. It is the capital city and one of the highly urbanized cities in the Kerala state of India. It is located in the coastal plains along the Lakshadweep Sea (Fig. 1). According to the Census 2011, the total geographical area of Thiruvananthapuram UA is 542.57 km^2^ and comprises 1,679,754 persons within its territory. It comprises one municipal corporation (M. Corp.), three municipalities (M), twenty-four census towns (CT), and two outgrowths (OG). It is considered a prominent educational and research hotspot, but gradually it has emerged as an information technology (IT) hub in the state and region. Thiruvananthapuram UA is under Seismic Zone III as a moderately earthquake-prone zone. It experiences a tropical savanna climate and monsoon climate where the mean maximum temperature is 34 °C, and the mean minimum temperature is 21 °C. During the monsoon season, the humidity is very high and rises to 90%.

For this study, secondary data sources, such as Landsat satellite images for the years 2001, 2007, 2014, and 2021, were downloaded from the USGS website (Table 1). Landsat satellite images with less than 10% cloud cover were selected to reduce the error. The details of urban agglomeration, such as total area, socioeconomic conditions, and UA boundary, were obtained from the Census of India 2011 website. ArcGIS 10.3 and MS Excel were used to quantify and monitor the spatial urban growth phenomenon in Thiruvananthapuram UA.

**Table 1:** Details of Landsat satellite imageries

S. No	Landsat sensor	Thiruvananthapuram UA				
		Scene ID	Path/row	No. of bands	Acquisition date	Grid gell size (m)
1	7 ETM	LE71440542001062SGS00	144 / 54	9	2001–03-03	30
2	5 TM	LT51430542007032BKT00	143 / 54	7	2007–02-01	30
3	8 OLI TIRS	LC81440542014026LGN01	144 / 54	9	2014–01-26	30
4	8 OLI TIRS	LC81440542021061LGN00	144 / 54	11	2021–03-02	30

## Methods

**Fig. 2:**
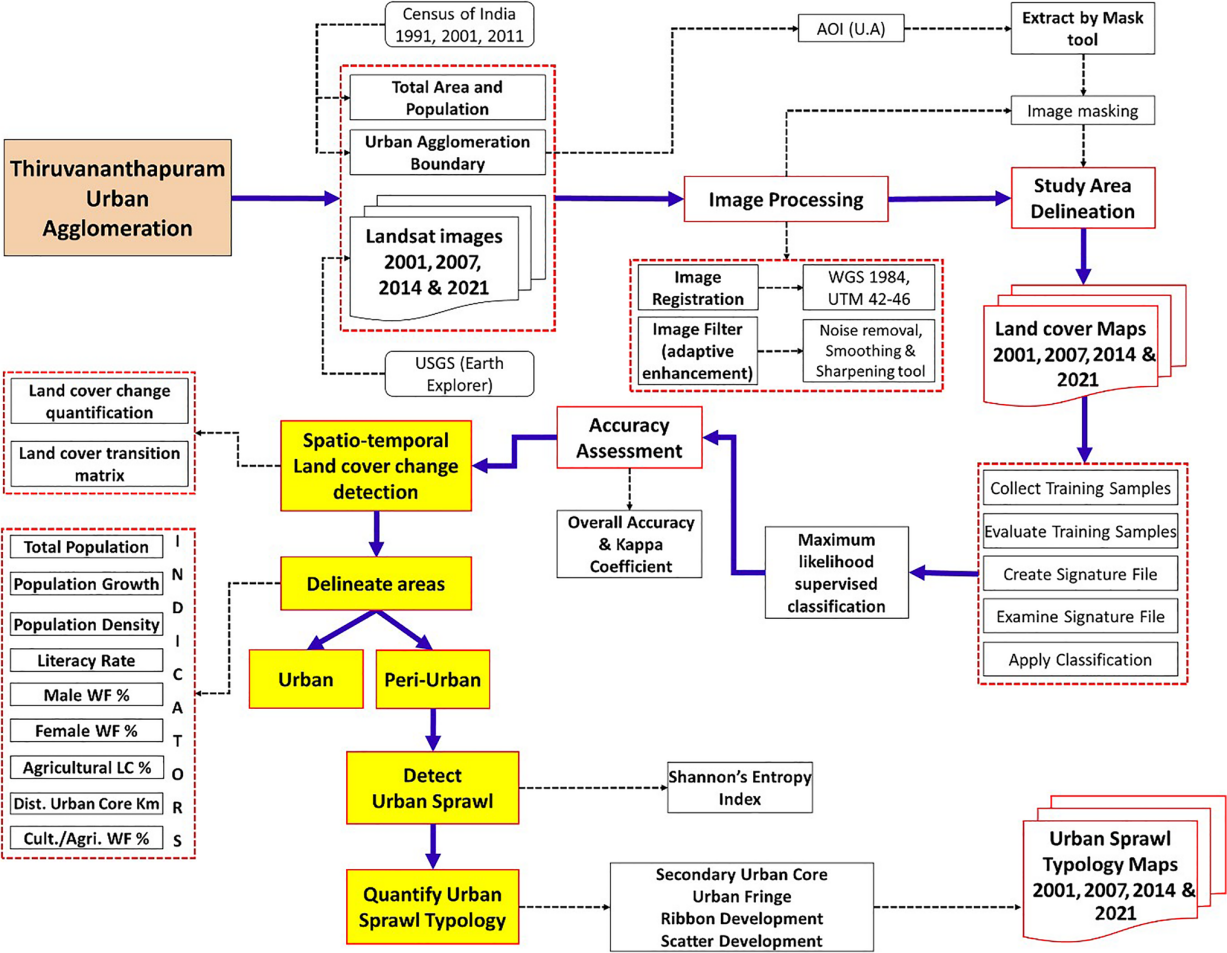
Methodology adopted in the study

The methodology adopted in this study to delineate the peri-urban areas and assess urban sprawl is exhibited in Fig. 2. Broadly, the study can be categorized into four stages: land cover change detection, peri-urban area delineation, urban sprawl detection, and quantification of urban sprawl typologies. Built-up land cover change detection computes the transformation of built-up land cover during the study period. The delineation of peri-urban areas was done using the nine indicators collected from the literature review. Shannon’s entropy index was used to detect urban sprawl, and lastly, the urban sprawl typologies in peri-urban areas were quantified. The details of each stage are discussed in the following subsections.

### Land cover change detection

The obtained satellite images of multiple periods were merged in ArcGIS 10.3 using the spectral tool. The merged image was preprocessed, which includes image registration and image enhancement for better land cover classification (Barow et al. 2019). The digitized UA boundary was used to mask the study area from the satellite images. The false-color composite (FCC) of the images was prepared, and using the maximum likelihood supervised classification (MLC) tool in ArcGIS 10.3, land cover maps were obtained (He et al. 2011; Zeng et al. 2015). It is based on the Bayes’ theorem (Eq. 1), in which the probability of the pixels depicting a particular land cover class is computed (Alkaradaghi et al. 2019).

1 $$P\left(A|B\right)=P\left(B|A\right)P\left(A\right)/P\left(B\right)$$

where *A* and *B* are events and *P(B) ≠ 0*; *P(A│B)* is the likelihood of event *A* occurring given that *B* is true, *P(B│A)* is the likelihood of event *B* occurring given that *A* is true, and *P(A)* and *P(B)* are the probabilities of observing *A* and *B* independently of each other.

The training samples for the MLC method were collected through visual image interpretation based on the color, form, size, tone, texture, and presence of shadows. The collected samples were evaluated, and a signature file was created for each period. Thereafter, the respective signature files were used to produce land cover maps for 2001, 2007, 2014, and 2021. The images were classified into five land cover classes, i.e., vegetation, built-up, waterbody, agriculture, and fallow land cover. However, this study focuses on urban growth analysis; therefore, land cover classes other than built-up were categorized as non-built-up. The accuracy of each land cover map was checked using ground truth points and verified through site visits and Google Earth (Moisa and Gemeda 2021). For both the land cover classes, i.e., built-up and non-built-up, hundred samples were selected based on the stratified random sampling method. Overall accuracy (*OA*) and Kappa coefficient (*k*_*i*_), as shown in Eqs. (2) and (3), determine the level of land cover accuracy (Sun et al. 2017; Liu et al. 2020).

2 $$OA=\frac{\sum_{i=1}^{r}{x}_{ij}}{n}$$

3 $$K=\frac{n\sum_{i=1}^{r}{x}_{ij}- \sum_{i=1}^{r}\left({x}_{i+}*{x}_{+i}\right) }{{n}^{2}- \sum_{i=1}^{r}\left({x}_{i+}*{x}_{+i}\right)}$$

where ${x}_{ij}=$ number of observations in row $i$ and column $j$ (along the major diagonal), $n=$ total number of samples in the error matrix, $r$ = number of rows in the error matrix, and ${x}_{i+}$ and ${x}_{+i}$ are the marginal totals of row $i$ and column $j$, respectively.

The overall accuracy is the ratio of total accurately categorized pixels to the total pixel numbers present on the map. According to the marginals found in the true classes, the Kappa coefficient determines how accurately a land cover classification is categorized than a random classification. The minimum accuracy value to achieve a satisfactory result is 85% (Siddiqui et al. 2018). Thereafter, built-up and non-built-up growth was quantified for the different study periods.

### Peri-urban areas delineation

The indicators selected to delineate peri-urban areas were referred from the literature (Salem, 2015; Chakraborti et al. 2018; Dutta and Das 2019a, b), and only 9 indicators were selected as per the data availability. It includes total population, population growth, population density, literacy rate, percentage of the male workforce, percentage of the female workforce, agricultural land cover, distance from urban core, and percentage of cultivators to agricultural workers. All the required values of the indicators were referred from the Census of India 2011 except the agricultural land cover, which was computed from the land cover map of 2014. The Statutory towns like M. Corp. and Municipality in Thiruvananthapuram UA were considered urban. The threshold value of each indicator used to classify urban and peri-urban areas is shown in Table 2. The administrative units are classified as urban areas if more than 75% of the indicators are satisfied as per the threshold values (urban), while the remaining are categorized as peri-urban areas.

**Table 2:** Indicators selected for the study to delineate peri-urban areas

S. No	Indicators	Threshold values of each indicator		Source
		Urban	Peri-urban	
1	Total population	≥ 5000	< 5000	(Census of India, 2011; Salem, 2015; Chakraborti et al. 2018; Dutta and Das 2019a, b)
2	Population growth (%)	≥ 20	< 20	
3	Population density (persons/km^2^)	≥ 400	< 400	
4	Literacy rate (% to the total population)	≥ 75	< 75	
5	Percentage of male workforce (%)	≥ 75	< 75	
6	Percentage of female workforce (%)	≥ 25	< 25	
7	Agricultural land cover (%)	≤ 50	> 50	
8	Distance from urban core (km)	≤ 15	> 15	
9	Cultivators to total agricultural workers (%)	≥ 50	< 50	

### Urban sprawl detection

Shannon’s entropy index was employed to detect urban sprawl (Feng et al. 2015). It is widely used in urban sprawl studies to quantify the compaction or dispersion of built-up areas. If the values approach zero, it designates concentrated development, and values closer to *log*_*e*_*(n)* designate urban sprawl (Gupta et al. 2018; Shukla and Jain 2019). In this study, the number of administrative units delineated as peri-urban areas was used to compute the value of *n*, and a variable *X* takes on a value *X*_*i*_ for any administrative unit, i.e., *(i* = *1,2,3…..n)*. Shannon’s entropy index *H*_*n*_ is calculated, as shown in Eqs. (4) and (5).

4 $$H_n=-\sum_{i=1}^nP\;i\mathrm{loge}(Pi)$$

5 $${P}_{i}={X}_{i}/\sum_{i=1}^{n}{X}_{i}$$

where *P*_*i*_ is the probability or the proportion of the variable occurring in the zone *i* and is calculated as shown in Eq. (5).

### Urban sprawl typologies

**Fig. 3:**
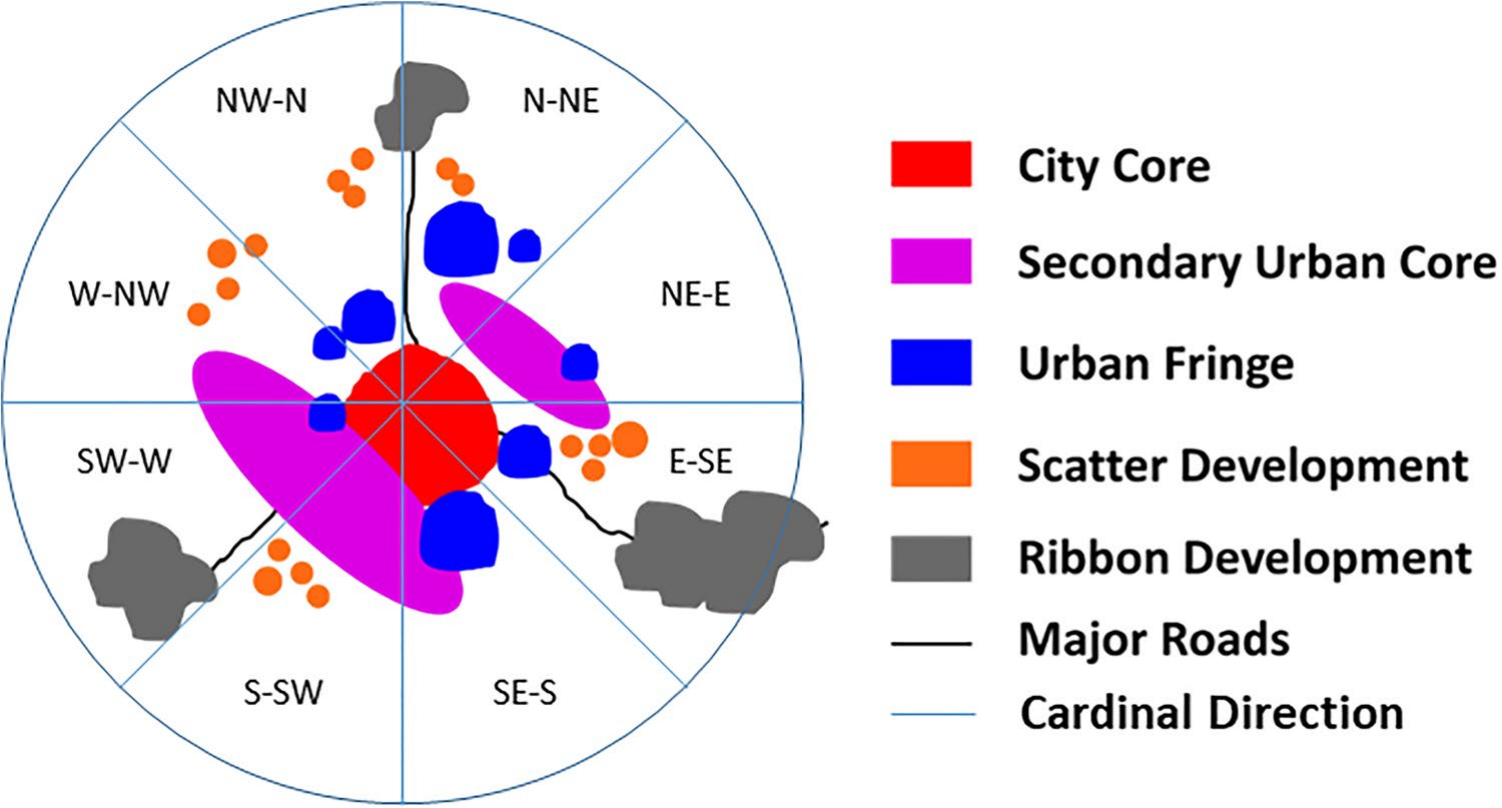
Schematic diagram of urban sprawl typologies

The urban sprawl typologies in the study area were computed using the adjacent neighborhood relationships concept. It is derived from the spatial heterogeneity of the surroundings that arises primarily due to the natural aspects such as topography; socioeconomic aspects, such as population and market factors; and risks such as floods and landslides. As per Chettry and Surawar (2021a, b, c), the urban sprawl types can be classified into four major categories such as secondary urban core, urban fringe, ribbon development, and scatter development (Fig. 3).

The secondary urban core is defined as the discontinuous dense built-up area with 80–50% of the built-up within 1-km^2^ neighborhood. Urban fringe is a discontinuous medium dense built-up area with 30–50% of the built-up within 1-km^2^ neighborhood. Similarly, scatter and ribbon development are characterized as a discontinuous low-dense built-up area with less than 30% of built-up within 1-km^2^ neighborhood, but later is computed only within the 100-m proximity to main routes such as national and state highways. For this analysis, the hexagon mesh of 1-km^2^ was used to classify the built-up area into four urban sprawl categories (Baltzis 2011). The threshold value of built-up in each hexagon cell is mentioned in Table 3 (Pașca and Năsui 2016; Masini et al. 2018).

**Table 3:** Details of urban sprawl typologies

S. No	Urban sprawl typologies	Threshold value	Description
1	Secondary urban core	80–50% built-up area in one sq.km	Discontinuous dense
2	Urban fringe	30–50% built-up area in one sq.km	Discontinuous medium dense
3	Scatter development	< 30% built-up area in one sq.km	Discontinuous low dense
4	Ribbon development	< 30% built-up area within 100 m proximity to national and state highways	

## Results

### Spatiotemporal land cover change detection

**Fig. 4:**
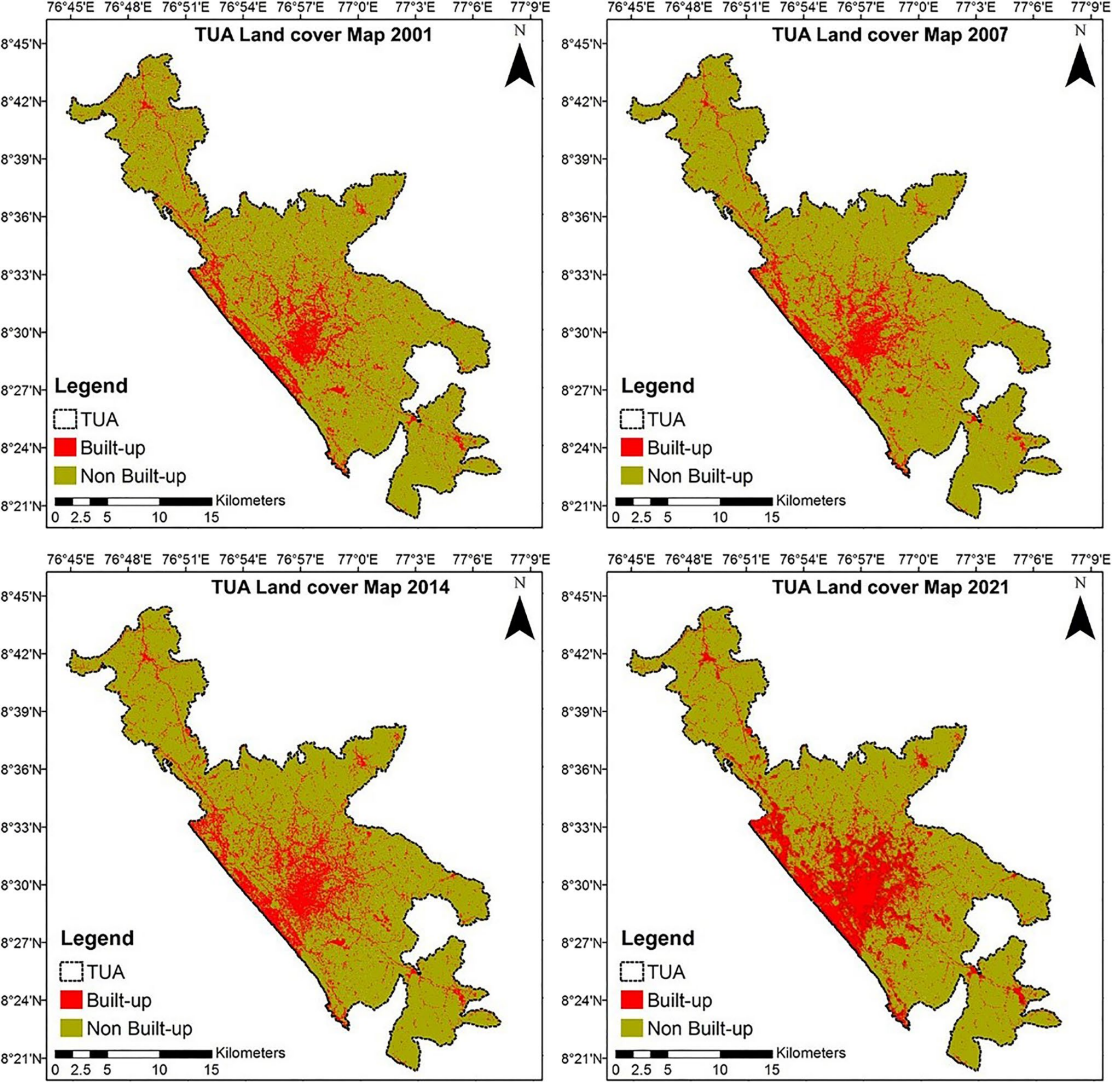
Land cover maps of Thiruvananthapuram UA in 2001, 2007, 2014, and 2021

The land cover maps of Thiruvananthapuram UA (TUA) in 2001, 2007, 2014, and 2021 obtained after the classification process are shown in Fig. 4.

This analysis helps to explore the notable transformation among the land cover classes. The accuracy of the land cover maps was assessed and exhibited in Table 4. The overall accuracy ranges from 90.7 to 92%, while the kappa coefficient ranges between 88 and 90.83%. Thus, the results obtained are satisfactory, and land cover maps can be used further for analysis.

**Table 4:** Accuracy assessment of land cover maps prepared for Thiruvananthapuram UA

S. No	Accuracy assessment	Thiruvananthapuram UA			
		2001	2007	2014	2021
1	Overall accuracy	90.7%	91.45%	91%	92%
2	Kappa coefficient	88%	89.04%	88.6%	90.83%

**Fig. 5:**
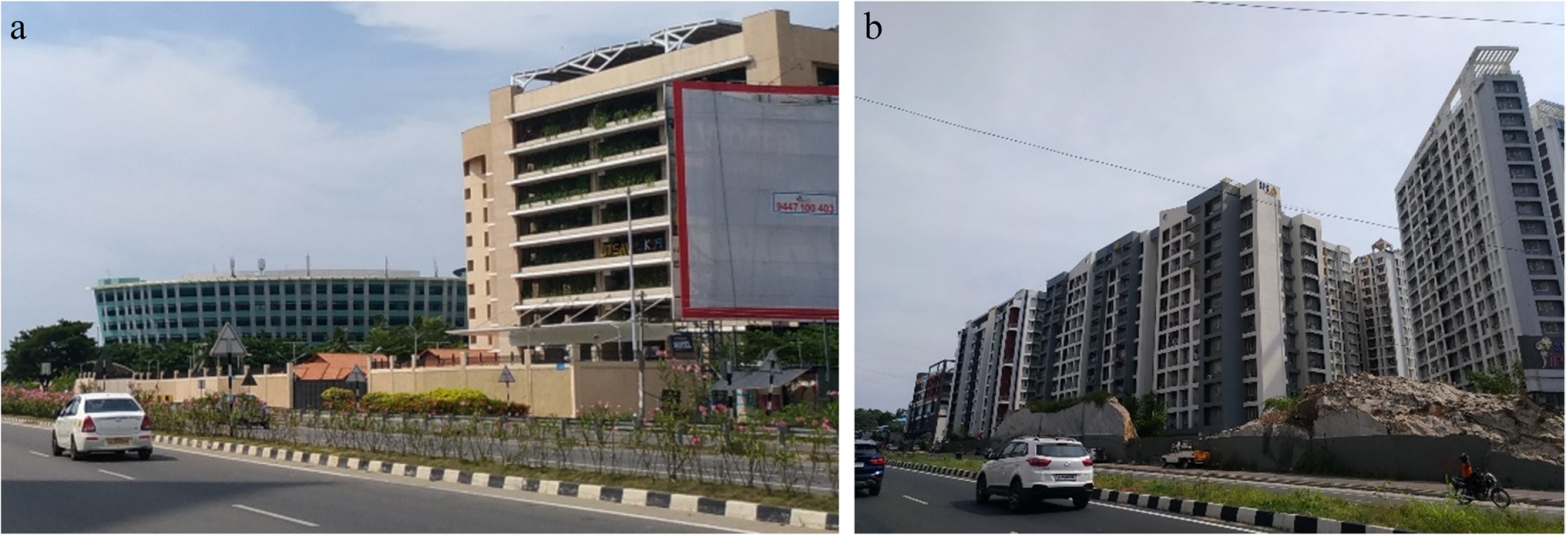
**a** Software technology parks in TUA, (**b**) Rising real estate market in TUA

From 2001 to 2021, the land cover transformation within TUA was quantified and exhibited in Table 5. During 2001–2021, the built-up land cover increased from 77.35 km^2^ (14%) in 2001 to 174.65 km^2^ (32%) in 2021 while non-built-up land cover has decreased from 465.22 km^2^ (86%) in 2001 to 367.92 km^2^ (68%) in 2021. Overall, the rate of built-up increase was 126%, and the rate of non-built-up decrease was − 21% from 2001 to 2021. Such rapid growth in built-up areas has mostly occurred within the Thiruvananthapuram (M. Corp), Kudappanakunnu (CT), Vattiyoorkavu (CT), Vilavoorkkal (CT), Kovalam (OG), Malayinkeezhu (CT), Kazhakkoottam (OG), and Pallippuram (CT). Most of this urban growth can be attributed to the rise in the real estate market and the set-up of service sector industries, i.e., IT software parks in the Kazhakkoottam (OG) and Pallippuram (CT) (Fig. 5a and b). Moreover, linear growth can be seen along the NH 47 connecting northern TUA, i.e., Attingal, to southern TUA, i.e., Neyyattinkara (M). NH 47 is a major road passing through the TUA, and it connects to other major cities such as Kochi, India, in the north direction and Kanyakumari, India, in the south direction.

**Table 5:** Land cover quantification in TUA during 2001, 2007, 2014, and 2021

S. No	Land cover classes	Land cover quantification from 2001 to 2021				% change 2001–2021
		2001	2007	2014	2021	
1	Built-up	77.35 km^2^	98.41 km^2^	123.78 km^2^	174.65 km^2^	126%
2	Non-built-up	465.22 km^2^	444.16 km^2^	418.79 km^2^	367.92 km^2^	-21%

### Delineation of peri-urban areas

**Fig. 6:**
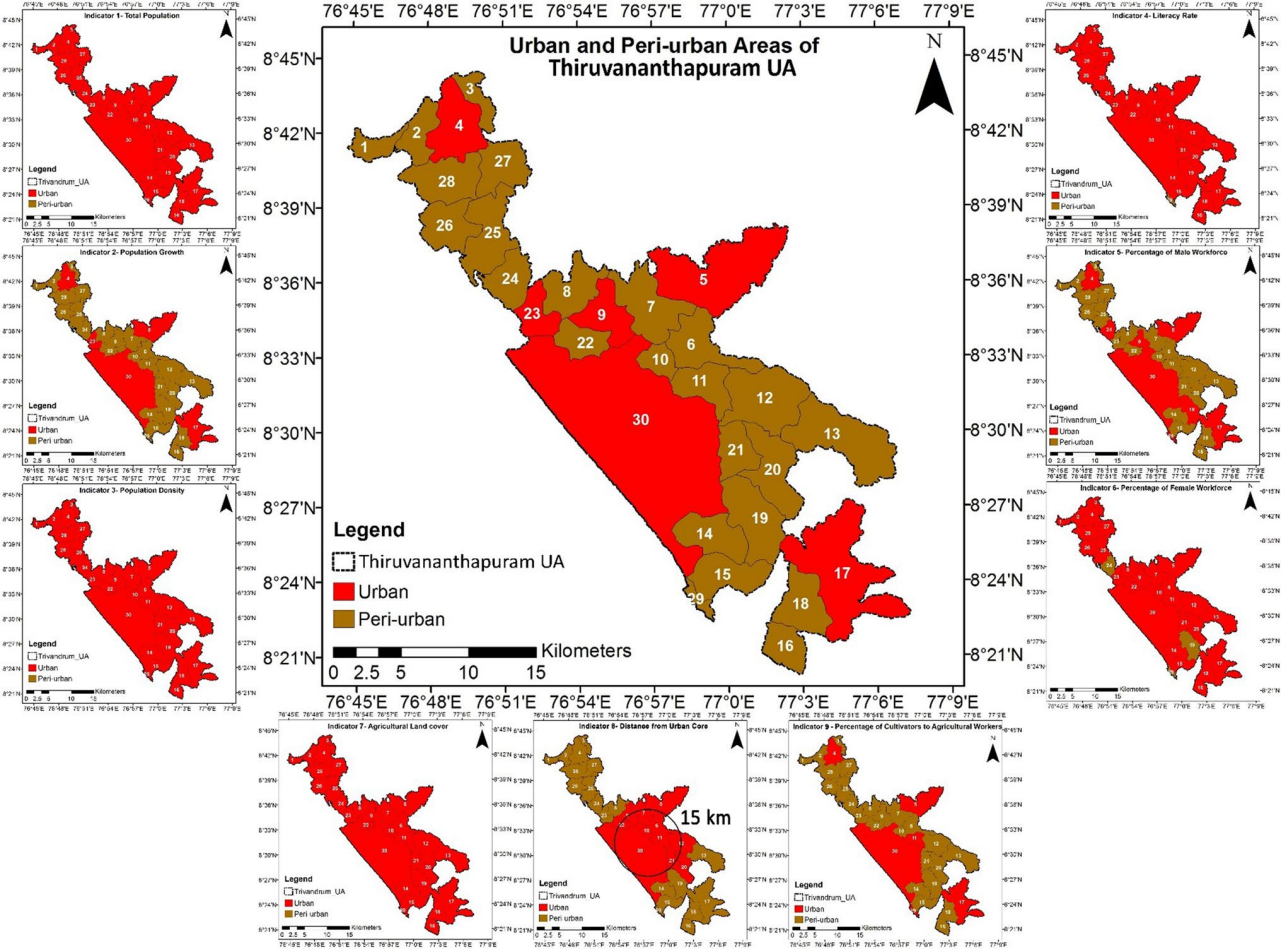
Delineation of peri-urban areas based on the selected nine indicators

A total of nine indicators were used to delineate urban and peri-urban areas of TUA. The delineated peri-urban areas based on the selected indicators are shown in Fig. 6. The urban areas included six, and peri-urban areas included twenty-four administrative units of TUA, as listed in Table 6. The total area under the urban category is 240.68 km^2^, i.e., 44.36%, while the peri-urban area is 301.89 km^2^, i.e., 55.64% of TUA. As per the Census 2011, the urban area encompasses a total population of 959,122 persons, and the peri-urban areas had 720,632 persons, i.e., 57.10% and 42.90%, respectively. The pattern of area and population distribution among the urban and peri-urban areas indicates that the total population is less than in the urban areas despite more areas being under the peri-urban category.

**Table 6:** List of administrative units delineated as urban and peri-urban areas

S. No	Typology of urban settlement	Administrative units	Area (km^2^)
1	Urban	Attingal, Kazhakkoottam, Nedumangad, Neyyattinkara, Thiruvananthapuram, and Uliyazhathura	240.68
2	Peri-urban	Alamcode, Athiyannur, Azhoor, Edakkode, Iroopara, Kalliyoor, Kanjiramkulam, Karakulam, Keezhattingal, Kizhuvalam-Koonthall, Kovalam, Kudappanakunnu, Kulathummal, Malayinkeezhu, Pallichal, Pallippuram, Sreekaryam, Vakkom, Vattappara, Vattiyoorkavu, Veiloor, Venganoor, Vilappil, and Vilavoorkkal	301.89

### Detection of urban sprawl

The delineated areas were investigated to detect urban sprawl using Shannon’s entropy index. The *log*_*e*_*(n)* value for this study is 3.17 (*n* = 24), and the overall urban sprawl values of peri-urban areas were computed during each study period. In 2001, the urban sprawl value was 1.59, which increased to 1.67 in 2007, 1.85 in 2014, and 2.05 in 2021. The lowest entropy value was obtained in 2001 at 1.59, indicating non-disperse development. The highest entropy value was obtained in 2021 at 2.05, indicating the occurrence of dispersed built-up growth, i.e., urban sprawl. Overall, the values are farther from 0 and towards *log*_*e*_*(n)*; thus, it indicates the occurrence of urban sprawl in the peri-urban areas of Thiruvananthapuram UA. The pattern of entropy index values from 2001 to 2021 suggests a rise in urban sprawl during the study period.

### Quantification of urban sprawl typologies

**Fig. 7:**
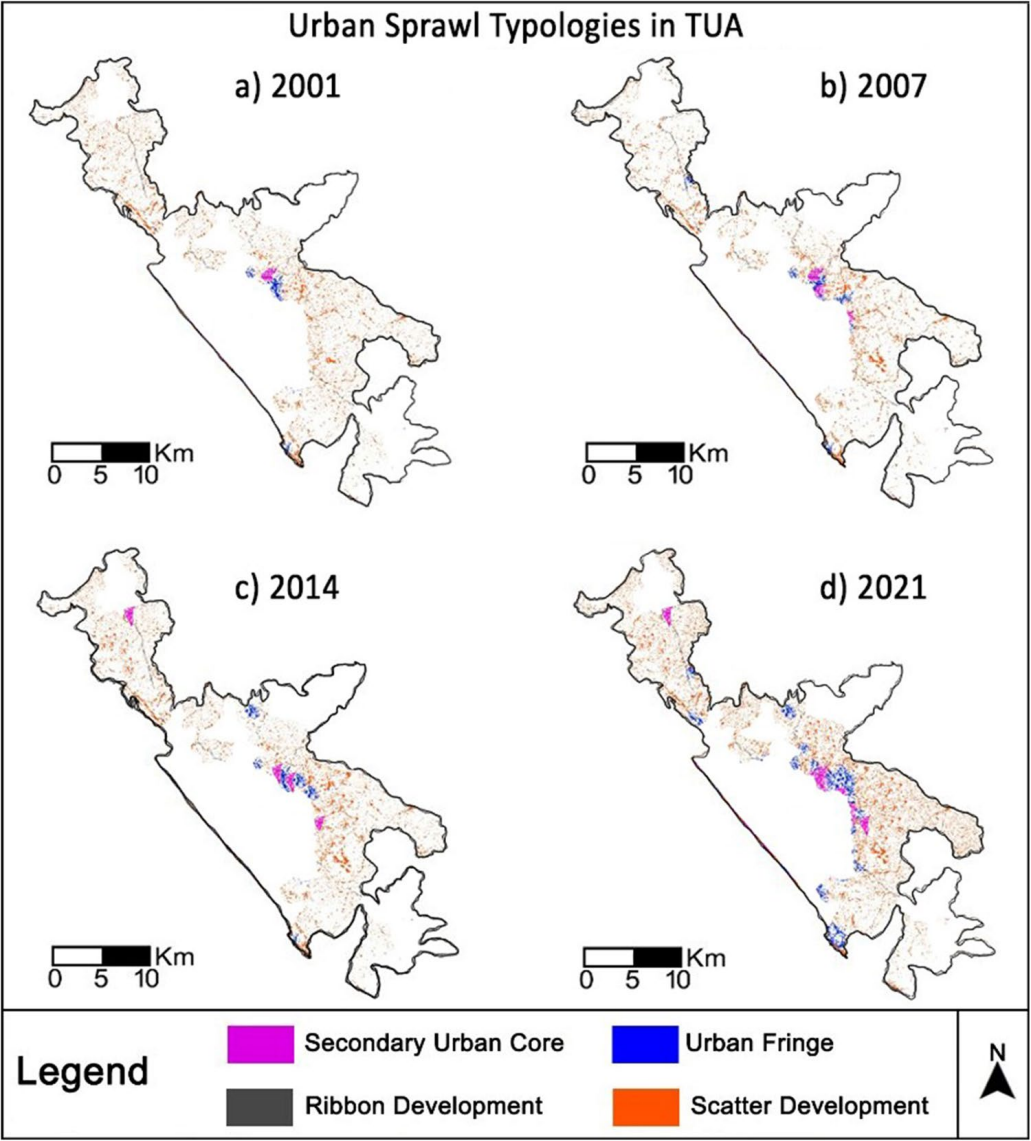
Urban sprawl typologies in TUA from 2001 to 2021

The quantification of urban sprawl typologies in the peri-urban areas revealed that in 2001, discontinuous dense development, i.e., the secondary urban core, was 0.84 km^2^; discontinuous medium dense development, i.e., urban fringe, was 1.89 km^2^; and discontinuous low-density development, i.e., scatter and ribbon development, was 27.90 km^2^ and 1.80 km^2^, respectively. In 2007, discontinuous dense development, i.e., the secondary urban core, was 1.43 km^2^; discontinuous medium dense development, i.e., urban fringe, was 3.98 km^2^; and discontinuous low-density development, i.e., scatter and ribbon development, was 30.62 km^2^ and 1.95 km^2^, respectively. In 2014, discontinuous dense development, i.e., the secondary urban core, was 5.31 km^2^; discontinuous medium dense development, i.e., urban fringe, was 4.76 km^2^; and discontinuous low-density development, i.e., scatter and ribbon development, was 34.82 km^2^ and 2.27 km^2^, respectively. In 2021, discontinuous dense development, i.e., the secondary urban core, was 8.96 km^2^; discontinuous medium dense development, i.e., urban fringe, was 6.64 km^2^; and discontinuous low-density development, i.e., scatter and ribbon development, was 39.81 km^2^ and 2.96 km^2^, respectively. Figure 7 exhibits the urban sprawl typologies observed in Thiruvananthapuram UA from 2001 to 2021.

The highest gain in urban sprawl typologies during 2001–2021 had occurred in scatter development, i.e., 11.91 km^2^, followed by secondary urban core typology (8.12 km^2^), urban fringe (4.75 km^2^), and ribbon development (1.16 km^2^). Overall scatter development was the dominant urban sprawl typology prevailing in the peri-urban areas of Thiruvananthapuram UA.

## Discussions

The land cover changes in Thiruvananthapuram UA from 2001 to 2021 were very rapid. There was considerable growth in built-up areas from 77.35 km^2^ in 2001 to 174.65 km^2^ in 2021. The rise in built-up areas was primarily through the reduction of non-built-up areas. Such rapid loss of non-built-up land cover poses a significant threat to the urban sustenance of the city. Similar characteristics of urban growth are visible in other midsized cities in India (Yatoo et al. 2020). Although Thiruvananthapuram acts as a service town, where people are primarily engaged in government and institutional activities, post-1990, there has been tremendous growth in industrial units within the UA. The establishment of Technopark at Kazhakootam in 1990 and KINFRA (Kerala Industrial Infrastructure Development Corporation) in 1993 developed small industries park at Thumba and a film and video park at Kazhakoottam (Thiruvananthapuram Corporation 2012; Chettry and Surawar 2021b). There is one industrial estate at Pappanamcode, one mini-industrial estate at Ulloor, and one industrial development center at Kochuveli. In the future, significant development is projected to occur around the Technopark phase II and Technopark phase III at Attinkuzhi. Vizhinjam International Deepwater Multipurpose Seaport at Vizhinjam would further drive the development of the UA. Due to the land crunch and rising real estate prices within the city core, these projects are planned in the peri-urban areas. Such a development pattern triggers urban sprawl and threatens urban sustenance.

Shannon’s entropy index confirmed the presence of urban sprawl in Thiruvananthapuram UA. The dominant typology of urban sprawl in the peri-urban areas was scatter development, indicating the prevalence of discontinuous low-density urban growth. The major cause of such development is due to dispersed pattern of settlement and industrial growth in the peri-urban areas (Arulbalaji et al. 2020). There is an absence of coordinating agencies for the census towns located in the periphery of Thiruvananthapuram UA. Moreover, there is lack of governing authority for UA in India. UA is delineated by the Census of India based on the criteria such as population and contiguity of nearby towns and villages surrounding any statutory towns. Although Thiruvananthapuram Development Authority (TRIDA) was constituted for the planned and scientific development of Thiruvananthapuram and its adjoining areas, the area under its jurisdiction is only 295.35 km^2^. Moreover, after the enactment of the 74th constitutional amendment act in 1992; the power and functions of TRIDA have been limited just to the implementation of small-scale projects. Hence, a metropolitan authority in Thiruvananthapuram is an urgent requirement to enhance coordination and promote sustainable development.

Overall, such urban growth phenomenon harms the local environment and causes irreversible changes in the natural landscape of the urban agglomeration. The nearby wetlands and agricultural fields are being utilized for development purposes. The effect of urban sprawl on the natural land covers was observed in other cities across the globe, which includes but are not limited to Dhaka (Dewan and Yamaguchi 2009); Shanghai (Yin et al. 2011), Nepal (Rimal et al. 2019); and Greater Cairo (Muhammad Salem et al. 2020). During the floods in 2018, Thiruvananthapuram UA was the least affected due to its undulating terrain, but the people residing in the encroached low-lying areas and wetlands were severely affected.

Recently the government of India has come up with a few policies which aim for sustainable development in peri-urban areas. National Rurban Mission (NRuM) was launched in 2016 to develop a cluster of villages by providing economic, social, and physical infrastructure facilities. National urban policy framework 2018 proposes the inclusion of peri-urban areas inside the planning area boundaries of cities. It also suggests the use of urban growth boundaries in accordance with regional resources, vulnerable ecosystems, and climatic considerations. Implementing these policies in peri-urban areas of Thiruvananthapuram UA would certainly promote urban sustainability.

## Conclusion

This study exhibited the application of geospatial techniques that utilized Landsat images and census data to investigate urban sprawl dynamics in peri-urban areas. Overall, this paper focused on land cover change assessment, delineation of peri-urban areas, detection of urban sprawl, and identification of urban sprawl typologies in peri-urban areas of Thiruvananthapuram UA.

Landsat satellite images (7ETM, 5TM, and 8OLI_TIRS) were downloaded for land cover change detection through MLC technique in ArcGIS 10.3. The land cover changes in the study area revealed the occurrence of rapid built-up growth primarily at the expense of non-built-up areas from 2001 to 2021. During the study period, the rise in built-up land cover was 126%, while non-built-up land cover declined by 21%. Such rapid and irreversible land cover changes in the research areas have created conflict between the environment and development. The peri-urban areas of the Thiruvananthapuram UA were delineated using 09 indicators as per the data availability, such as total population, population growth, population density, literacy rate, percentage of the male workforce, percentage of the female workforce, agricultural land cover, distance from the urban core, and percentage of cultivators to agricultural workers. The total area under the urban category is 240.68 km^2^, i.e., 44.36%, while the peri-urban area is 301.89 km^2^, i.e., 55.64% of Thiruvananthapuram UA. Shannon’s entropy index values for peri-urban areas exhibited a rise in urban sprawl. Further, the quantification of urban sprawl typologies revealed the prevalence of scatter development, i.e., discontinuous low-density development in Thiruvananthapuram UA.

Urban planners and concerned authorities can use it as a future reference for the planned urban growth in Thiruvananthapuram UA. However, the lack of high-resolution satellite images and unavailability of other important datasets to delineate peri-urban areas (% people who received high education, average income, and unemployment rate) remains a limitation of this study. The census survey in India for the year 2021 could not be conducted due to the pandemic (COVID-19); hence, the delineation of peri-urban areas in Thiruvananthapuram UA was based on 2011 data. The future research scope can include the usage of machine learning techniques to forecast urban growth in the delineated peri-urban areas.
